# Rupture Risk Assessment for Anterior Communicating Artery Aneurysms Using Decision Tree Modeling

**DOI:** 10.3389/fcvm.2022.900647

**Published:** 2022-05-13

**Authors:** Jinjin Liu, Haixia Xing, Yongchun Chen, Boli Lin, Jiafeng Zhou, Jieqing Wan, Yaohua Pan, Yunjun Yang, Bing Zhao

**Affiliations:** ^1^Department of Radiology, The First Affiliated Hospital of Wenzhou Medical University, Wenzhou, China; ^2^Department of Neurosurgery, Renji Hospital, Shanghai Jiao Tong University School of Medicine, Shanghai, China; ^3^Department of Pathology, Shanghai East Hospital, Tongji University School of Medicine, Shanghai, China; ^4^Department of Nuclear Medicine, The First Affiliated Hospital of Wenzhou Medical University, Wenzhou, China

**Keywords:** intracranial aneurysm, anterior communicating artery aneurysm, rupture risk, decision tree model, machine learning

## Abstract

**Background:**

Although anterior communicating artery (ACoA) aneurysms have a higher risk of rupture than aneurysms in other locations, whether to treat unruptured ACoA aneurysms incidentally found is a dilemma because of treatment-related complications. Machine learning models have been widely used in the prediction of clinical medicine. In this study, we aimed to develop an easy-to-use decision tree model to assess the rupture risk of ACoA aneurysms.

**Methods:**

This is a retrospective analysis of rupture risk for patients with ACoA aneurysms from two medical centers. Morphologic parameters of these aneurysms were measured and evaluated. Univariate analysis and multivariate logistic regression analysis were performed to investigate the risk factors of aneurysm rupture. A decision tree model was developed to assess the rupture risk of ACoA aneurysms based on significant risk factors.

**Results:**

In this study, 285 patients were included, among which 67 had unruptured aneurysms and 218 had ruptured aneurysms. Aneurysm irregularity and vessel angle were independent predictors of rupture of ACoA aneurysms. There were five features, including size ratio, aneurysm irregularity, flow angle, vessel angle, and aneurysm size, selected for decision tree modeling. The model provided a visual representation of a decision tree and achieved a good prediction performance with an area under the receiver operating characteristic curve of 0.864 in the training dataset and 0.787 in the test dataset.

**Conclusion:**

The decision tree model is a simple tool to assess the rupture risk of ACoA aneurysms and may be considered for treatment decision-making of unruptured intracranial aneurysms.

## Introduction

Unruptured intracranial aneurysms (IAs) are increasingly detected with the development of modern imaging modalities (1), such as magnetic resonance imaging angiography and CT angiography (CTA). Anterior communicating artery (ACoA) aneurysms are the most common IAs, accounting for approximately 30% (2). Although aneurysms located at ACoA have a higher risk of rupture than those located in other locations (3), whether to treat unruptured ACoA aneurysms is still a dilemma because treatment-related complications still exist (4). This dilemma further brings considerable anxiety to these patients with unruptured IAs.

Morphologic features, hemodynamics parameters, and genetic factors for aneurysm rupture have been widely reported (5, 6). A literature review (7) has shown that size ratio, the direction of the dome, and fenestration were the independent predictors of ACoA aneurysm rupture. Our previous study has shown a larger aneurysm, anterior projection of the dome, the dominant A1 segment, and irregular aneurysms were associated with aneurysm rupture (8). However, these results are inconsistent probably because the relationship between these morphologic parameters and aneurysm rupture is complex (5). Accurately assessing the rupture risk of IAs is still a challenging task.

Machine learning is capable of finding the nonlinear complex relationship between input and output variables and has been applied in the medical field (9). Machine learning models, such as support vector machines, artificial neural networks, and random forests have been applied for the prediction of rupture risk of IAs (10–13). As a supervised machine learning technique, decision tree modeling can provide a visualized graph including a set of rules for predictive classification (14), which satisfies the easy-to-use requirement in clinics.

In this study, we performed a retrospective analysis of rupture risk for patients with ACoA aneurysms. Morphologic parameters of these aneurysms were measured and evaluated. We aimed to assess the rupture risk of ACoA aneurysms using decision tree modeling.

## Materials and Methods

### Patients

This study was approved by local institutional ethics committees and written informed consent was waived. We retrospectively reviewed patients with ACoA aneurysms at the First Affiliated Hospital of Wenzhou Medical University from December 2007 to January 2016 and at Renji Hospital, Shanghai Jiao Tong University School of Medicine from March 2017 to October 2019. We excluded patients with fusiform ACoA aneurysms because fusiform aneurysms are rare and have different underlying pathologies, hemodynamics, natural histories, and treatments compared to saccular aneurysms. We also excluded patients with multiple ACoA aneurysms, patients with Moyamoya disease or arteriovenous malformations, and patients with a brain tumor. Those with poor image quality were also excluded to ensure measurement accuracy of aneurysm morphology. Patients’ demographic and clinical information, including sex, age, history of smoking, and hypertension were retrieved from medical records. All aneurysms were grouped into ruptured and unruptured groups according to the clinical condition.

### Aneurysm Morphologic Parameters

Aneurysm morphologic parameters were measured or evaluated on CTA or digital subtraction angiography by independent neuroradiologists who were blind to patients’ clinical information. The average value was used for data analysis. Corresponding imaging technique was published elsewhere (15).

**FIGURE 1:**
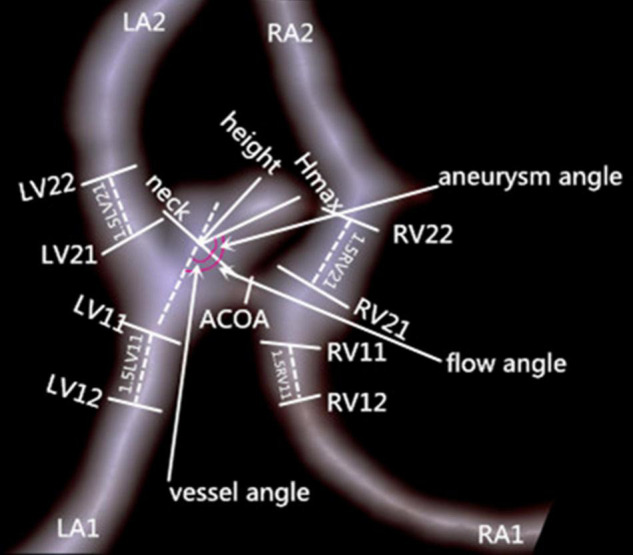
Measurement of morphologic parameters. Left ventricle (LV)11 and LV12 represent cross-section diameters of the artery, left atrium (LA)1 proximal to aneurysm neck and at 1.5XLV11 away from aneurysm neck, respectively; vessel size of LA1 is calculated as (LV11+LV12)/2. Vessel sizes of LA2, right atrium (RA)1, and RA2 are similarly calculated. Hmax is aneurysm height.

Figure 1 shows the measurement of morphological parameters. Detailed definitions of these parameters are summarized as follows: (1) aneurysm size, maximal aneurysm diameter; (2) vessel size, mean cross-sectional diameter of all arteries associated with an aneurysm; (3) aneurysm height, largest distance from the center of aneurysm neck to aneurysm dome; (4) perpendicular height, largest perpendicular distance from the center of aneurysm neck to aneurysm dome; (5) neck size, largest neck diameter; (6) aspect ratio, ratio between perpendicular height and neck size; (7) size ratio, aneurysm height divided by vessel size; (8) aneurysm angle between aneurysm neck line and aneurysm height line; (9) vessel angle, angle between the vector of blood flow and aneurysm neck line; and (10) flow angle, the angle between aneurysm height line and vector of blood flow in the parent artery. Aneurysm irregularity was classified into three types, i.e., regular, bleb, and daughter-sac (16). Projection of the aneurysm dome was dichotomized as anterior and posterior projections. A1 segment configurations were classified into symmetrical, dominant, and complete configurations according to the inflow contribution of A1 segments over the other A2 segments (17).

### Decision Tree Modeling

We partitioned data into the training and test datasets with a ratio of 80:20. Features were selected with a correlation-based feature subset selection method using the training dataset in Weka 3.8.5 (The University of Waikato, Hamilton, New Zealand). Grid search strategy with a fivefold cross-validation was used to acquire the optimal hyper-parameters, and the following parameters were obtained: criterion, Gini impurity; maximum depth of the tree, 4; minimum number of samples to split, 10; minimum number of samples required to be at a leaf node, 5; the number of features to consider, 7; class weight, “balanced.” Note that Gini impurity or Gini index measures the probability of incorrectly classifying an element randomly chosen in the dataset if it is randomly labeled according to the class distribution in the dataset. The decision tree iteratively splits current data into two categories during training, and Gini index quantitatively evaluates the quality of a split. The equation of the Gini index is (18).

$$\text{Gini}=\sum_{i=1}^{n}p_{i}\,\left(1-p_{i}\right),$$

where *p*_*i*_ is the probability of an object being classified to a particular class. The Gini index varies from 0 to 1, where 0 denotes that all elements belong to a certain class or if there exists only one class and is the best possible impurity, 0.5 means that elements are equally distributed into some classes, and 1 represents those elements which are randomly distributed across various classes.

We evaluated model performance using sensitivity, specificity, accuracy, and area under the receiver operating characteristic (ROC) curve (AUC). Sensitivity is the fraction that a model prediction will indicate ruptured aneurysms among those with ruptured aneurysms; specificity measures the probability of those with unruptured aneurysms who will have a model prediction result of unruptured aneurysms.

### Statistical Analysis

We performed statistical analysis using the software SPSS 22 (IBM Corp, Armonk, NY, United States). We presented continuous variables as mean value ± *SD* and categorical variables as frequency (percentage). We compared continuous variables using the Student’s *t*-test or Mann–Whitney U tests, and categorical variables using Fisher exact test or χ^2^ tests, as appropriate. We further conducted a multivariate logistic regression analysis to determine the independent risk factors of aneurysm rupture, and those variables with *P*-value less than 0.1 were entered into the analysis (variables with missing values more than 5% were excluded). *P*-values less than 0.05 were considered statistically significant.

## Results

### Baseline Characteristics

Table 1 shows patients’ baseline characteristics. Two hundred and eighty-five patients were enrolled in this study. Of these patients, 136 (47.7%) were female and 149 (42.3%) were male, with a mean age of 58.2 ± 11.8 years. Among them, 67 had unruptured aneurysms and 218 had ruptured aneurysms. Forty-one patients had multiple aneurysms. Patients with unruptured aneurysms were significantly more likely to be older (61.8 ± 9.8 vs. 57.1 ± 12.1 years).

**TABLE 1:** Baseline characteristics.

	All (*n* = 285)	Unruptured (*n* = 67)	Ruptured (*n* = 218)	*P*-value
Sex (women)	136 (47.7%)	34 (50.7%)	102 (46.8%)	0.571
Age (years)	58.2 ± 11.8	61.8 ± 9.8	57.1 ± 12.1	0.001
Smoking (yes) ^a^	71 (24.9%)	14 (20.9%)	57 (26.1%)	0.685
Hypertension (yes)^a^	152 (53.3%)	39 (58.2%)	113 (51.8%)	0.580
Multi aneurysms (yes)	41 (14.4%)	14 (20.9%)	27 (12.4%)	0.083

*^a^24 (8.4%) missing values.*

### Morphologic Characteristics Between Ruptured and Unruptured Anterior Communicating Artery Aneurysms

Table 2 illustrates the comparison of morphological parameters between ruptured and unruptured aneurysms. The shape of ruptured aneurysms was more frequently irregular (bleb or daughter-sac types) (41.3 vs. 16.4%), whereas unruptured aneurysms tended to be regular (83.6 vs. 58.7%). Anterior projection of an aneurysm dome was more common in ruptured aneurysms than in unruptured ones (71.7 vs. 55.2%). Aneurysm size, vessel size, aneurysm height, neck size, and size ratio were significantly larger in ruptured aneurysms than in unruptured ones. Aneurysm angle was significantly larger in unruptured aneurysms, while vessel and flow angles were obviously larger in ruptured aneurysms.

**TABLE 2:** Morphological parameters between ruptured and unruptured aneurysms.

	All (*n* = 285)	Unruptured (*n* = 67)	Ruptured (*n* = 218)	*P*-value
Aneurysm size (mm)	5.11 ± 2.63	4.24 ± 2.50	5.37 ± 2.62	0.002
Vessel size (mm)	1.97 ± 0.48	2.08 ± 0.47	1.94 ± 0.47	0.042
Aneurysm height (mm)	4.14 ± 1.35	3.39 ± 2.22	4.37 ± 2.35	0.003
Perpendicular height (mm)	3.32 ± 1.80	2.96 ± 1.94	3.43 ± 1.75	0.066
Neck size (mm)	3.05 ± 1.20	2.77 ± 1.12	3.14 ± 1.21	0.026
Aspect ratio	1.15 ± 0.59	1.15 ± 0.70	1.15 ± 0.56	0.985
Size ratio	2.24 ± 1.46	1.71 ± 1.21	2.40 ± 1.50	<0.001
Aneurysm angle (°)	67.81 ± 18.29	71.56 ± 18.63	66.66 ± 18.07	0.028
Vessel angle (°)	57.20 ± 30.30	42.98 ± 30.56	61.57 ± 28.91	<0.001
Flow angle (°)	133.53 ± 29.15	121.90 ± 28.26	137.10 ± 28.54	<0.001
Aneurysm irregularity				<0.001
Regular type	184 (64.6%)	56 (83.6%)	128 (58.7%)	
bleb type	61 (21.4%)	3 (4.5%)	58 (26.6%)	
Daughter-sac type	40 (14.0%)	8 (11.9%)	32 (14.7%)	
Aneurysm projection				0.015
Anterior	192 (67.4%)	37 (55.2%)	155 (71.7%)	
Posterior	93 (32.6%)	30 (44.8%)	63 (28.9%)	
A1 segment configuration				0.045
Symmetric A1 segment	124 (43.5%)	38 (56.7%)	86 (39.4%)	
Dominant A1 segment	82 (28.8%)	15 (22.4%)	67 (30.7%)	
Absent A1 segment	79 (27.7%)	14 (20.9%)	65 (29.8%)	

Results of multivariate logistic regression to assess rupture risk of ACoA aneurysms are presented in Table 3. Vessel angle and aneurysm irregularity were the independent predictors of aneurysm rupture.

**TABLE 3:** Results of multivariate logistic regression analysis.

Variables	β coefficient	OR	95% CI	*P*-value
Vessel angle	0.02 ± 0.01	1.02	1.01–1.03	<0.001
Aneurysm irregularity				
Regular type		1.0 (reference)		
Bleb type	1.99 ± 0.62	7.31	2.17–24.68	0.001
Daughter-sac type	0.65 ± 0.44	1.92	0.82–4.54	0.140

*OR, odds ratio; CI, confidence interval.*

### Decision Tree Model

**FIGURE 2:**
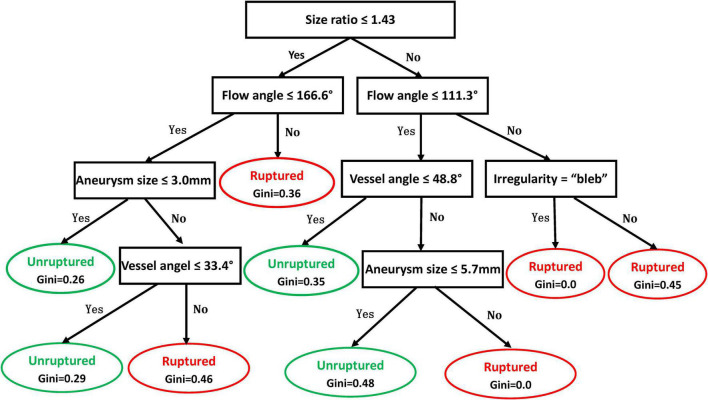
Decision tree model for rupture risk assessment of anterior communicating artery (ACoA) aneurysms. In the decision tree, rectangle nodes represent conditions and elliptical nodes stand for the ruptured or unruptured status of the aneurysm predicted. One can start from the root node (i.e., “Size ratio ≤ 1.43”) and compare the value of the size ratio of an aneurysm with the not node. If it is true that the size ratio is less or equal to 1.43, then the next node of “Flow angle ≤ 166.6°” can be moved; otherwise, jump to the node of “Flow angle 111.3°.” Continue comparing the attribute value of an aneurysm with other internal nodes of the decision tree until an elliptical node is reached, at which point the predicted status, ruptured or unruptured, is obtained.

Illustrated in Figure 2 is the decision tree model for rupture risk assessment of ACoA aneurysms. The model used 5 variables for risk assessment, including size ratio, flow angle, vessel angle, aneurysm size, and aneurysm irregularity. A detailed explanation of how to use the decision tree is shown in the legend of Figure 2. Continue comparing the attribute value of an aneurysm with other internal nodes of the decision tree until an elliptical node is reached, at which point the predicted status, rupture, or unruptured, is obtained.

**FIGURE 3:**
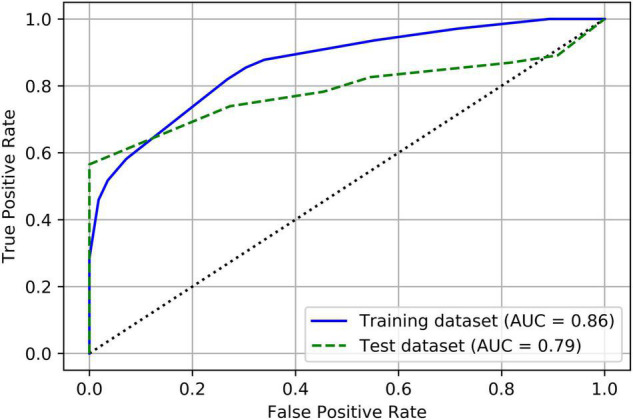
Receiver operating characteristic (ROC) curves of the decision tree model for both training and test datasets.

Table 4 summarizes the prediction results of the decision tree model. In the training dataset, the model achieved a sensitivity of 82%, a specificity of 73.2%, and an overall accuracy of 79.8%. In the test dataset, the model achieved a sensitivity of 73.9%, a specificity of 72.7%, and an overall accuracy of 73.7%. Figure 3 shows the ROC curves of the decision tree model for both training and test. AUC were 0.864 and 0.787 for the training and test datasets, respectively.

**TABLE 4:** Prediction results of aneurysm rupture.

Actual class	Predicted class		
	Unruptured	Ruptured	Accuracy (%)
(a) Training dataset			
Unruptured	41	15	73.2
Ruptured	31	141	82.0
Overall			79.8
(b) Test dataset			
Unruptured	8	3	72.7
Ruptured	12	34	73.9
Overall			73.7

*AUC, area under the curve.*

## Discussion

In this study, we measured detailed morphologic features of ACoA aneurysms and investigated their association with aneurysm rupture. Aneurysm irregularity and vessel angle were independent predictors of rupture of ACoA aneurysms. Size ratio, aneurysm irregularity, flow angle, vessel angle, and aneurysm size were selected for decision tree modeling. An easy-to-use decision tree model achieved a good performance in assessing the rupture risk of ACoA aneurysms.

Although many morphologic parameters contribute to aneurysm rupture, aneurysm size is the most common one to assess the rupture risk of unruptured aneurysms (19, 20). A meta-analysis of a large number of aneurysms demonstrated that rupture risk of aneurysms increased with increased aneurysm size (21). The Unruptured Cerebral Aneurysm in Japan (UCAS) cohort found a similar trend (3). Population, hypertension, age, size of an eurysm, earlier subarachnoid hemorrhage, site of a neurysm (PHASES) scoring system (19) used aneurysm size as one of the significant predictors of aneurysm rupture. Except for aneurysm size, we found aneurysm irregularity and vessel angle were the independent predictors of rupture of ACoA aneurysms. Aneurysm irregularity represents the shape regularity of aneurysms. Several studies (3, 22) have found a significant correlation between aneurysmal shape and rupture risk. Irregular aneurysms are more commonly found in ruptured aneurysms than in unruptured aneurysms (23). One of the predictors for aneurysm growth in the ELAPSS score system was the shape of an aneurysm (24). Dhar et al. (25) proposed vessel angle for the first time. They evaluated vessel angles between 25 unruptured and 20 ruptured intracranial aneurysms and found no significant difference; however, Zheng et al. (26) reported a contrasting result. We found vessel angle was significantly larger in ruptured aneurysms than in unruptured aneurysms, which was consistent with the result of Zheng et al. Vessel angle incorporates the parent vessel geometry and implies blood flow direction, which may reflect hemodynamic characteristics (26, 27).

We developed a model combining the valuable morphologic parameters to assess the rupture risk of ACoA aneurysms. The PHASES score (28) used several risk factors to evaluate aneurysm rupture risk, and only one morphologic feature (i.e., aneurysm size) was considered. Recent studies (4, 29) found that this score might only provide a weak tool for evaluating aneurysm rupture risk and more parameters beyond those in the PHASES score might be needed to improve prediction performance. Another popular score consisted of six predictors, ELAPSS (24), which used two morphologic features, aneurysm size, and shape, for predicting the risk of growth of IA. This ELAPSS score was further externally validated and showed accurate calibration and modest discrimination in the external validation cohort (30). Therefore, more significant morphologic parameters may provide additional valuable information for rupture risk evaluation of IAs. We included aneurysm size, size ratio, aneurysm irregularity, flow angle, and vessel angle to develop a decision tree model to assess the rupture risk of ACoA aneurysms.

The decision tree model is capable of finding complex nonlinear relationships between variables (14). We developed the decision tree model achieving a relatively good prediction performance with overall accuracies of 79.8% in the training dataset and 73.7% in the test dataset by combining five valuable morphologic variables. This model is a tree-like structure that shows the various outcomes from a series of decisions, which consists of three main elements: a root node, leaf nodes, and branches. Any path beginning from the root node is described by a data separating sequence until a Boolean outcome at the leaf node is achieved (31). Currently, with the advancement of machine learning techniques, support vector machines, artificial neural networks, linear, ridge, and lasso regression models, and random forest have been applied for rupture risk assessment of IAs (10–13). The random forest models of Tanioka et al. (13) achieved accuracies of 77, 71.2, and 78.3% by using morphologic parameters, hemodynamic parameters, and both morphologic and hemodynamic parameters, respectively. Accuracy was not significantly improved by adding hemodynamic features, and possible reasons were that scientists used generalized boundary conditions instead of patient-specific boundary conditions. Compared with other machine learning methods, the decision tree visually demonstrates cause-and-effect relationships and provides a simplified and easy-to-understand view of a potentially complicated process (14). Therefore, our finding suggests that the decision tree model may be an ideal tool to assess aneurysm rupture.

There are several limitations to this study. First, this is not an observational prospective natural history study of aneurysms [such as the ISUIA study (20)], which may limit the application of our decision tree model in future rupture risk assessment of IAs. Second, IAs may shrink after rupture, which may influence the measurement accuracy of morphologic parameters. However, several studies found no evidence for shrinkage of IAs after rupture (32, 33). Third, our model has not been externally validated. Finally, only the Chinese population is involved in this study. Previous studies have found that the rupture risk of IA is population-dependent. The generalization of our model to other populations should be with caution.

## Conclusion

In summary, we investigated risk factors associated with ACoA aneurysm rupture and developed a decision tree model to assess rupture risk based on size ratio, flow angle, vessel angle, aneurysm irregularity, and aneurysm size. Our model achieved a good performance and is easy to use, which may facilitate the decision-making of treatment for unruptured ACoA aneurysms.

## Data Availability Statement

The raw data supporting the conclusions of this article will be made available by the authors, without undue reservation.

## Ethics Statement

The studies involving human participants were reviewed and approved by the Ethical Committee of Renji Hospital and the First Affiliated Hospital of Wenzhou Medical University. Written informed consent for participation was not required for this study in accordance with the national legislation and the institutional requirements.

## Author Contributions

JL, YY, and BZ were involved in the conceptualization of the study. JL, HX, YC, BL, JZ, JW, YP, and BZ were involved in the acquisition and analysis of the data. JL and BZ contributed to the statistical analysis and model development and wrote the first manuscript. YY and BZ were guarantors of the overall content. All authors were involved in data interpretation, read, and approved the final manuscript.

## Conflict of Interest

The authors declare that the research was conducted in the absence of any commercial or financial relationships that could be construed as a potential conflict of interest.

## Publisher’s Note

All claims expressed in this article are solely those of the authors and do not necessarily represent those of their affiliated organizations, or those of the publisher, the editors and the reviewers. Any product that may be evaluated in this article, or claim that may be made by its manufacturer, is not guaranteed or endorsed by the publisher.
